# Nest Material Shapes Eggs Bacterial Environment

**DOI:** 10.1371/journal.pone.0148894

**Published:** 2016-02-12

**Authors:** Cristina Ruiz-Castellano, Gustavo Tomás, Magdalena Ruiz-Rodríguez, David Martín-Gálvez, Juan José Soler

**Affiliations:** Departamento de Ecología Funcional y Evolutiva, Estación Experimental de Zonas Áridas (CSIC), Almería, Spain; University of Lleida, SPAIN

## Abstract

Selective pressures imposed by pathogenic microorganisms to embryos have selected in hosts for a battery of antimicrobial lines of defenses that includes physical and chemical barriers. Due to the antimicrobial properties of volatile compounds of green plants and of chemicals of feather degrading bacteria, the use of aromatic plants and feathers for nest building has been suggested as one of these barriers. However, experimental evidence suggesting such effects is scarce in the literature. During two consecutive years, we explored experimentally the effects of these nest materials on loads of different groups of bacteria (mesophilic bacteria, *Enterobacteriaceae*, *Staphylococcus* and *Enterococcus*) of eggshells in nests of spotless starlings (*Sturnus unicolor*) at the beginning and at the end of the incubation period. This was also explored in artificial nests without incubation activity. We also experimentally increased bacterial density of eggs in natural and artificial nests and explored the effects of nest lining treatments on eggshell bacterial load. Support for the hypothetical antimicrobial function of nest materials was mainly detected for the year and location with larger average values of eggshell bacterial density. The beneficial effects of feathers and plants were more easily detected in artificial nests with no incubation activity, suggesting an active role of incubation against bacterial colonization of eggshells. Pigmented and unpigmented feathers reduced eggshell bacterial load in starling nests and artificial nest boxes. Results from artificial nests allowed us to discuss and discard alternative scenarios explaining the detected association, particularly those related to the possible sexual role of feathers and aromatic plants in starling nests. All these results considered together confirm the antimicrobial functionality mainly of feathers but also of plants used as nest materials, and highlight the importance of temporally and geographically environmental variation associated with risk of bacterial proliferation determining the strength of such effects. Because of costs associated to nest building, birds should adjust nest building effort to expected bacterial environments during incubation, a prediction that should be further explored.

## Introduction

Bird nests are infected by numerous parasites that affect dramatically their reproductive output [1–3]. The best known are nest-dwelling ectoparasites, like mites and fleas [4,5]. Microorganisms are also common in nests [6], some of them being highly pathogenic for developing embryo [7]. They can cross the eggshell [8], cause diseases in embryos [9] and, thus, reduce egg viability [10]. However, eggs have numerous defensive traits against pathogens like the eggshell and antimicrobial contents [11–15]. Although costly immunological barriers of eggs are quite effective fighting off potential pathogens [16,17], parents have also evolved additional defensive mechanisms to maintain their eggs free of parasites and pathogenic bacteria. For instance, birds can modulate their incubation behaviour in order to reduce humidity and thus conferring protection from precipitation or water that favour bacterial penetration [18,19].

Some other birds like hoopoes (*Upupa epops*) preen their eggs with their own uropygial gland secretions to reduce density of pathogenic bacteria on the eggshell [3,20,21]. Others build a new–free of parasites–nest every year [6], or remove the old nest materials from cavities before breeding [22,23]. Some other bird species use substances produced by other animal or plant species for protection against pathogens (self-medication; [24,25]).

A type of self-medication is the use of nest material with antimicrobial properties [26]. There are numerous materials used by birds with antimicrobial properties among which cigarette butts has been recently added to the list [27]. The most studied nest materials with known antipathogenic effects are green plants [1,28,29]. Most of the used green plants are aromatic plants that contain volatile compounds or essential oils [1], which can play a repellent, fumigant or toxic role reducing abundance or minimizing the effect of pathogenic bacteria [30–32] and parasites [28,33]. Experimental evidence on antimicrobial properties of green plants reducing risk of bacterial infection on developing nestlings [30–32] and embryos [34] is however scarce.

Nest lining feathers have traditionally been studied for their thermoregulatory properties [35,36] or their function as sexual display [37–40]. More recently, evidence of an antimicrobial function has been found in barn swallow nests (*Hirundo rustica*) [41,42]. This function may be due to bacterial strains, like *Bacillus licheniformis*, that live on feathers and digest the keratin (the main component of feathers), and are able to outcompete other bacteria by producing antibiotic agents [14,43]. Those antimicrobials can help to fight off other bacteria with potentially stronger negative effects on developing embryos and nestlings. It is even known that the antimicrobial properties of bacteria degrading pigmented and unpigmented feathers differ depending on the nest lining feather composition [44]. Thus, the effects on the nest bacterial environment would depend on the abundance of pigmented and unpigmented feathers lining the nest of birds [14]. Evidences of the antimicrobial benefits of feathers are only known for barn swallow nests [41,42,44], and exploring the expected effect on nests of other species is needed.

Some avian species such as blue tits (*Cyanistes caeruleus*) or spotless starlings (*Sturnus unicolor*) use both green plants and feathers as nest material [31,38,45]. Since antimicrobials of plants may affect not only pathogenic, but also antibiotic-producing bacteria of feathers, an interaction between both materials explaining bacterial environment of nests may be expected; a hypothesis that has not been hitherto investigated. In addition, since incubation activity can affect bacterial environment of nests (i.e., reducing eggshell bacterial load, e.g., [18,19,46]), this behaviour may also modulate the effect of nest materials on bacterial density on eggshells. Thus, taking into account the effects of incubation is crucial to explore the isolate effect of nest materials on eggshell bacterial loads.

Here, we tried to fill these gaps with a study in spotless starlings, a species in which adults introduce green plants and feathers during the nest building and incubation stages. The use of feathers and green plants as nest material acts as sexual signals [37,45], and here we explore the possibility of additional antimicrobial functionality. Experimentally, we explore the combined effect of feathers and green plants explaining eggshell bacterial load in presence and absence of incubation. Each natural and artificial nest was randomly assigned to one of three feather treatments (only unpigmented feathers, only pigmented feathers or without feathers) and to one of two aromatic plants treatments (with or without aromatic plants). These experiments were performed in two different years and in different areas. Because of the presumed antibacterial effects of plants and feathers, we expected a reduced eggshell bacterial load in nests where either plants or feathers were included. We also expected an interacting effect between experimental treatments because the antimicrobial compounds of plants may clean beneficial bacteria of feathers. Moreover, we expected that the effects of antimicrobial compounds should be more easily detected in high density bacterial environments (i.e., years or areas where the highest bacterial density were detected).

## Material and Methods

### Ethics statement

The study was performed according to relevant Spanish national (Decreto 105/2011, 19 de Abril) and regional guidelines. The protocol was approved by ethics committee of Spanish National Research Council (CSIC) and all necessary permits for nest and egg manipulations were obtained from Consejería de Medio Ambiente de la Junta de Andalucía, Spain (Ref: SGYB/FOA/AFR/CFS and SGMN/GyB/JMIF). Our study area is not protected but privately owned, and the owners allowed us to work in their properties. This study did not involve endangered or protected species.

Time spent in each starling nest was the minimum necessary for bacterial sampling and for treatment application. This experiment did not show detectable effects in adult incubation behaviour or egg viability.

### Study area and field work

The study was performed during the breeding seasons 2012 and 2013 in Hoya de Guadix, southeast Spain (37°18’N, 3°11’W), a high-altitude plateau 1000m a.s.l, with a semi-arid climate. There were 80 cork-made nests boxes (internal height * width * depth: 350 * 180 * 210mm, bottom-to-hole height: 240mm) available for spotless starlings attached to tree trunks or walls at 3-4m above ground. Our starling population usually commences to build their nests in March and they use green plants and feathers as nest material. Green plants and feathers are embedded in the nests, forming part of both their structural and lining layer. Our starling population laid eggs at mid-April, and since April 10^th^ we visited nest boxes every three days until the first egg was laid. Laying dates were later in 2012 than in 2013 (2012: 27.45 ± 0.96; 2013: 23.00 ± 0.90 (April 1^st^ = 1); ANOVA: F = 11.24, df = 1,115, P = 0.001). Incubation period in spotless starlings starts before the clutch is finished, usually with the third or fourth egg, and lasts for 7–12 days after laying the third egg. Environmental conditions in our study area differed between years. Mean daily temperatures, as well as minimum and maximum temperatures, were higher in 2012 (14.7 ± 0.9°C, 7.8 ± 0.7°C and 21.7 ± 1.1°C) than in 2013 (11.9 ± 0.7°C, 6.3 ± 0.6°C and 18.3 ± 0.9°C) (ANOVA: F = 6.20, df = 1,76, P 0.015, F = 2.56, df = 1,76, P = 0.114, and F = 6.27, df = 1,76, P = 0.015, respectively). Total rainfall during the laying period was higher in 2013 (36.8mm) than in 2012 (25.2mm). Thus, mean humidity was higher in 2013 (70.89 ± 2.10%) than in 2012 (49.41 ± 2.51%) (ANOVA: F = 43.43, df = 1,76, P < 0.001) (data was obtained from the nearest climatological station, sited in Jerez del Marquesado: http://www.juntadeandalucia.es/agriculturaypesca/ifapa/ria/servlet/FrontController?action=Static&url=coordenadas.jsp&c_provincia=18&c_estacion=6).

### Experimental procedures

#### Preparation of experimental nest lining feathers and aromatic plants

We collected white (i.e., unpigmented) and non-white (i.e., pigmented) body feathers of similar size as those used by starlings as lining material from chickens that grew in small farms close to the study area, which are common nest materials used by starlings in our population. Feathers were sterilized in the laboratory using a UV sterilizer chamber (Burdinola BV-100) during 10 minutes on each feather side. Subsequently, to homogenize the bacterial load and colonies on feathers, we sprayed, with an atomizer, approximately 84ml of a high concentration solution of *Bacillus licheniformis* D13 per m^2^ of surface completely covering experimental feathers. Solution was made from an overnight growth of a *Bacillus* colony in 6ml of BHI (Brain Heart Infusion) media at 37°C on an orbital shaker. Finally, in separate hermetic bags we stored 15 pigmented or unpigmented feathers (i.e., the average number of feathers found in starling nests in previous years in the study area) at 4°C until its use in experimental nests. We used *Bacillus licheniformis* because it is a common feather-degrading and antimicrobial-producing bacterium [14].

Plants introduced in nests were a mixture of the four plant species most used by starlings in our population (personal observation); *Marrubium vulgare*, *Artemisia barrelieri*, *Lamium amplexicaule* and *Anacyclus clavatus*. All these plants have volatile compounds or essential oils with known antimicrobial activity [47–50]. Fragments of plants of similar size as those used by starlings were collected the same day of the experiment in the surroundings of the study area and therefore were placed fresh in nests in the nest cup, below the eggs. We weighed 1.7g of plant mixture for each nest (approx. 0.425g of each plant species) because this is the maximum amount of green plants that we found in starling nests in previous years in the study area (personal observation).

#### Experimental design in natural nests

**Fig 1:**
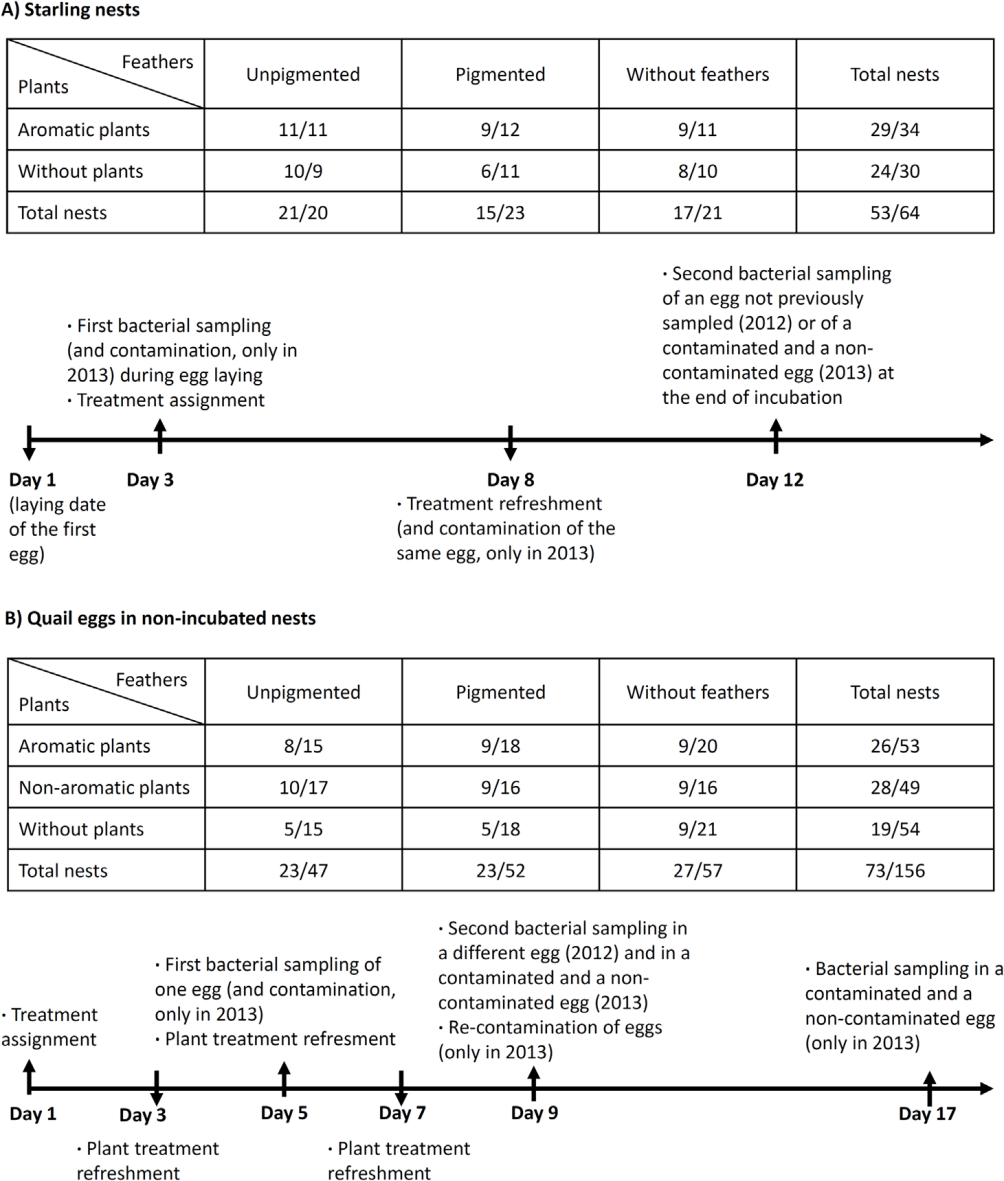
Experimental design of artificial and natural nests. Experimental protocols designed for exploring the effects of feathers and aromatic plants as nest materials on bacterial loads on spotless starling eggs (A) and on quail eggs in non-incubated nests (B). Numbers within the tables indicate sample sizes of different experimental treatment for 2012/2013 study years.

Our experiment followed a factorial design with feathers and aromatic plants (Fig 1A). Feathers treatments consisted of allocating (i) 15 pigmented, or (ii) 15 unpigmented feathers to the nest, or (iii) leave the nest without feathers. Plants treatments consisted of (i) introducing 1.7g of a mixture of aromatic plants or (ii) leave the nest without plants. Our experiment started on day 3 (i.e., nests had three eggs), by removing all plants and feathers that starlings had visible in their nests. Each nest was assigned to each of the experimental treatments for feathers and plants (Fig 1A, see below). On day 3, we also numbered each egg with a permanent marker (Staedtler permanent Lumocolor), and, before experimental manipulation, we sampled the eggshell of an egg to characterize the nest bacterial environment at the beginning of the experimental treatment. On day 8 (i.e., at the beginning of incubation), we remarked each egg and measured length and breadth of all eggs in nests with a digital caliper to the nearest 0.01mm. We also counted nest-lining feathers (distinguishing between pigmented and unpigmented), removed those added by adults, and refreshed the experimental treatment by adding pigmented and unpigmented feathers up to achieve the initial numbers. Finally, we removed and weighted green plants that were added by adults to nest materials and refreshed the experimental treatment. On day 12 (i.e., at the end of incubation), we sampled again the eggshells of one egg per nest that was not sampled during the first visit to characterize the bacterial environment of the nest after the experimental treatment. We also counted all lining feathers in the nest. A reliable estimation of green plant weight was not possible because small pieces were included as lining material on nest cup but also inserted within the nest material, being impossible to extract them without affecting nest structure. Thus we did not quantify green material.

We collected information on eggshell bacterial loads for 117 starling nests, 53 in 2012 and 64 in 2013 (see S1 Appendix). The experiments were effective in causing differences between nests under different treatments in the number of pigmented and unpigmented feathers at the time of hatching (Table 1).

**Table 1:** Influence of experimental treatments on nest lining feathers and aromatic plants.

	(A) NEST LINING FEATHERS							
		TREATMENTS						
		No feathers	Pigmented	UnPigmented	Statistical tests			
Year		Mean, SE (N)	Mean, SE (N)	Mean, SE (N)	F	df	P	Tolerance
2012	Number of feathers	19.0, 3.3 (17)	21.7, 10.9 (15)	23.2, 2.6 (21)	1.18	2.50	0.315	0.955
	Pigmented feathers	9.9, 1.6 (17)	14.3, 1.8 (15)	10.4, 2.5 (21)	2.50	2,50	0.092	0.909
	**Unpigmented feathers**	**9.1, 2.2 (17)**	**7.5, 1.5 (15)**	**12.8, 1.4 (21)**	**4.61**	**2,50**	**0.015**	**0.844**
2013	Number of feathers	23.6, 3.0 (21)	27.5, 3.2 (23)	26.2, 3.3 (20)	0.35	2,61	0.703	0.989
	**Pigmented feathers**	**3.1, 0.7 (21)**	**10.0, 0.9 (23)**	**2.3, 0.8 (20)**	**25.25**	**2,61**	**0.000**	**0.547**
	Unpigmented feathers	20.5, 2.9 (21)	17.4, 2.7 (23)	24.0, 2.9 (20)	2.46	2,61	0.094	0.727
	(B) AROMATIC PLANTS							
		TREATMENTS						
		No aromatic plants	Aromatic plants		Statistical tests			
Year		Mean, SE (N)	Mean, SE (N)		F	df	P	Tolerance
2012	Number of feathers	19.1, 2.3 (24)	23.4, 2.3 (29)		1.51	1,51	0.224	0.971
	Pigmented feathers	10.4, 2.1 (24)	12.1, 7.5 (29)		1.26	1,51	0.268	0.976
	Unpigmented feathers	8.7, 1.1 (24)	11.3, 1.6 (29)		0.65	1,51	0.423	0.970
2013	Number of feathers	29.7, 2.9 (30)	22.3, 2.1 (34)		3.36	1,62	0.072	0.949
	Pigmented feathers	6.0, 1.1 (30)	4.7, 0.8 (34)		0.16	1,62	0.699	0.997
	Unpigmented feathers	23.7, 2.7 (30)	17.7, 1.9 (34)		0.63	1,62	0.428	0.990

Influences of (A) feathers treatment (pigmented, unpigmented and without feathers) (B) and aromatic plants treatment (with or without) on nest lining feathers found in spotless starling nests at the end of incubation. Statistical tests were performed with log-transformed variables. Significant P-values are in bold.

#### Experimental design in artificial nests

The experimental design for artificial nests was similar to that of natural nests. We included an additional treatment to the aromatic plant experiment consisting of adding 1.7g of green barley (*Hordeum vulgare*) leaves (i.e., a non-aromatic plant, see [51]) as a control of aromatic plants.

This experiment was performed at two different localities in each of the two study years (Pinos (i.e., Area 1) and Pocico (i.e., Area 2) in 2012 and Calahorra (i.e., Area 3) and Area 1 in 2013). These areas are relatively close to each other and belong to Hoya de Guadix area. 73 new nest-boxes in 2012 and 156 in 2013 were placed in the study area (see S1 Appendix) for this experiment. The entrance of these nest-boxes was closed with a plastic mesh to prevent birds´ and/or predators´ access. They were filled (one fourth of the volume) with previously ultraviolet sterilized polyester fiberfill, on top of which experimental nest material (aromatic/non aromatic plants and/or feathers) were placed in a hollow simulating a nest cup. Two and three quail eggs previously cleaned with disinfectant wipes (Aseptonet, LaboratoiresSarbec, Cod.998077-51EN) were laid on top of the experimental nest material in 2012 and 2013 respectively.

Experiments in nest-boxes with no incubation activity were all performed the same day (the 2^nd^ of April in 2012 and the 11^th^ of April in 2013, hereafter day 1) (Fig 1B). Nest-boxes were visited every second day to refresh aromatic and non-aromatic plants. Also, every second day the eggs were gently moved ensuring contact of the whole eggshell surface with nest lining material. Bacteria from shells of each egg were sampled only once. Thus, different experimental eggs were sampled on day 5 and on day 9. In 2013, we collected a third sample on day 17.

#### Contamination experiment procedure

Only in 2013, we performed an additional experiment consisting on infecting starling and quail eggshells with bacterial strains known to be able to cross avian eggshells (Fig 1). These bacteria were collected from the interior of hen eggs that were kept in nest-boxes in the study area for two-three weeks (i.e., were exposed to the environmental conditions of the study area). Briefly, with a sterile rayon swab (EUROTUBO® DeltaLab) wet with a solution of 300μl of sodium phosphate buffer (0.2M; pH7.2) and 300μl of egg contents with bacteria (we confirmed a high bacterial load in this solution by overnight cultivation at 37°C), we besmeared two starling and three quail eggs in two-thirds of the nests under different plants’ and feathers’ experimental treatments leaving the other nests as controls.

#### Bacterial sampling

For each nest visiting and sampling we wore new gloves sterilized with 96% ethanol to prevent contamination of eggshell bacterial samples among nests. For eggshell bacterial sampling we cleaned the complete egg surface with a sterile rayon swab (EUROTUBO® DeltaLab) slightly wet with sterile sodium phosphate buffer (0.2M; pH = 7.2). After cleaning, we introduced the swab in an Eppendorf tube with the buffer solution and preserved it at 4–6°C in a portable refrigerator until being processed in the laboratory within 24h after collection.

### Laboratory work

After vigorously shaken in a vortex (Boeco V1 Plus), eggshell bacterial samples of starlings and quails were cultivated in four different solid media (Scharlau Chemie S.A., Barcelona). For that, we spread homogeneously 100μl of serially diluted samples until 10^−6^.

We used Tryptic Soy Agar, a broadly used general medium to grow aerobic mesophilic bacteria, and three specific media: Hektoen Enteric Agar for *Enterobacteriaceae*, Vogel-Johnson Agar for *Staphylococcus*, and Kenner Fecal Agar for *Enterococcus*. Plates were incubated at 37°C and after 72h the number of colonies on each plate was counted. For more details see [41].

Eggshell bacterial density was estimated by standardization of the number of colonies per cm^2^ of sampled eggshell (CFU, Colony Forming Units). Eggshell surface was estimated following Narushin formula [52] from length and width of each egg (S = 3*L^0.771^*W^1.229^, where S is the egg surface in cm^2^, W is the egg width and L is the egg length). Characterization of bacterial environments by traditional culture techniques have been demonstrated as appropriate for exploring associations between eggshell bacterial density and risk of embryo infection [10,18,21] and, thus, for our purposes.

### Sample sizes and statistical analysis

Mesophilic bacteria and number of feathers did approximately follow normal distributions after log10 transformation. The effects of feather and plant treatments on mesophilic bacterial loads and growth during the incubation period (i.e., changes in bacterial load between sampling events) were separately analyzed for different study years by means of General Linear Models (GLM). Experimental treatments were included as fixed discrete factors, and the following variables as continuous predictors: (i) date of sampling, log-transformed (ii) number of pigmented and (iii) unpigmented feathers at the time of experimental manipulation, and (iv) number of pigmented and (v) unpigmented feathers found in starling nests soon before hatching. Including the number of feathers at the end of incubation together with experimental treatments does not imply collinearity problems because of relatively low correlation coefficient among these two factors (Table 1) [53].

Contrary to one of our predictions, the interaction between treatments was far from statistical significance for all models tested (P > 0.2) and, thus, it was not considered for the final analyses. Non-significant terms in the models with the highest p-value were removed one by one up to p-values lower than 0.1. Results are shown for both complete (in Appendices) and reduced statistical models.

Prevalence of bacteria growing in specific media was relatively low in starlings eggshells (*Enterobacteriaceae*: 9.7% and 3.7%; *Staphylococcus*: 7.5% and 10.4%; and *Enterococcus*: 14.9% and 10.4% for first and second sampling, respectively). Consequently, we analyzed presence/absence rather than density in relation to experimental treatments in Generalized Linear Models (GLZ) with binomial error and logic link function. Factors in these models were those included in GLM models explaining mesophilic bacterial loads without log-transformation and the analyses controlled for overdispersion. In the model of first sampling, effects of nest material are shown for the complete model, because we did not detected any effect of the experiment. In the other models non-significant terms with the highest p-value were removed in the same way than for GLM. Chi-square Maximum Likelihood values were estimated in a type III analysis.

Prevalence of specific bacteria on non-incubated nest boxes was very low (< 2% in all cases) and, thus, the effects of experimental treatments on eggshell bacterial loads and on probability of trans-shell colonization were analyzed only for mesophilic bacteria. Since all experimental boxes were explored the same day and the whole nest lining material was experimental (i.e., no covariable that varied among sampling date was necessary), we explored these effects in Repeated Measures ANOVAs.

All statistical tests were performed with Statistica 8.0 (Statsoft Inc).

## Results

### Eggshell bacterial loads in natural starling nests

#### Nest material and mesophilic bacterial loads comparisons between first and second sampling

In first sampling, at time of egg laying pigmented feathers were more abundant in 2012 than in 2013, whereas unpigmented and total number of feathers did not differ between study years (Table 2). Density of mesophilic bacteria on the eggshell was higher in 2013 than in 2012 breeding season (Table 2).

**Table 2:** Among years variation in nest feathers and bacteria.

	2012		2013		Comparisons	
	Mean (SE)	N	Mean (SE)	N	F	p
Laying (day 3)						
log number of feathers	2.243 (0.121)	53	2.074 (0.094)	64	1.25	0.266
**log pigmented feathers**	**1.715 (0.136)**	**53**	**1.195 (0.121)**	**64**	**8.21**	**0.005**
log unpigmented feathers	1.371 (0.124)	53	1.517 (0.091)	64	0.93	0.336
**log mesophilic bacterial density**	**0.994 (0.079)**	**53**	**1.302 (0.062)**	**64**	**9.69**	**0.002**
End of incubation (day 12)						
log number of feathers	2.973 (0.074)	53	3.162 (0.063)	64	3.80	0.054
**log pigmented feathers**	**2.264 (0.102)**	**53**	**1.420 (0.126)**	**64**	**25.71**	**<0.0001**
**log unpigmented feathers**	**2.173 (0.103)**	**53**	**2.832 (0.098)**	**64**	**21.25**	**<0.0001**
**log mesophilic bacterial density**	**1.016 (0.056)**	**53**	**1.277 (0.051)**	**64**	**12.00**	**0.001**
Along incubation changes (Δ day 3-day 12)						
**log number of feathers**	**0.730 (0.141)**	**53**	**1.088 (0.112)**	**64**	**4.05**	**0.047**
log pigmented feathers	0.549 (0.164)	53	0.224 (0.181)	64	1.71	0.194
**log unpigmented feathers**	**0.801 (0.146)**	**53**	**1.315 (0.121)**	**64**	**7.48**	**0.007**
log mesophilic bacterial density	0.022 (0.082)	53	-0.025(0.064)	64	0.21	0.649

Inter-annual differences in nest lining materials (total, pigmented and unpigmented feathers) and density of mesophilic bacteria on spotless starling eggshells during the laying stage, at the end of the incubation period and changes experienced during the incubation period. Significant p-values are in bold.

In second sampling, at the end of incubation, density of mesophilic bacteria and number of unpigmented feathers were higher in samples from 2013 than in those from 2012 (Table 2). Number of pigmented feathers were however lower in 2013 than in 2012 (Table 2).

#### Nest material and experimental treatments effect on mesophilic bacterial load

**Fig 2:**
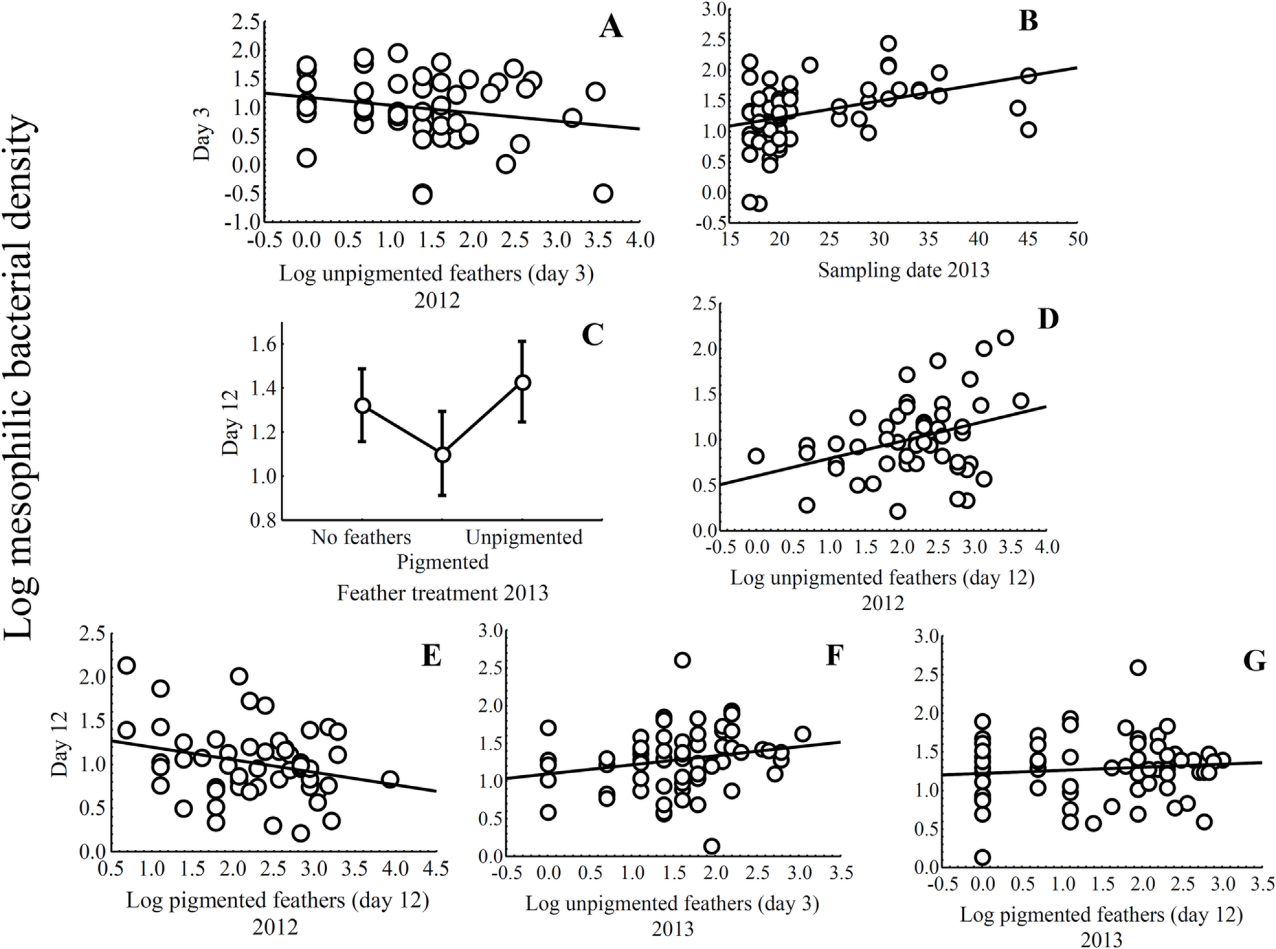
Nest material and experimental treatments effect on mesophilic bacterial load. Statistically significant relationships between loads of mesophilic bacteria of starling eggshells at day 3 in relation to number of unpigmented feathers in 2012 (A) and date of sampling in 2013 (B). The relationships between eggshell mesophilic bacterial load at day 12 (± 95% CI) in relation to feather treatment in 2013 (C), unpigmented feathers at day 12 in 2012 (D), pigmented feathers at day 12 in 2012 (E), unpigmented feathers at day 3 in 2013 (F) and pigmented feathers at day 12 in 2013 (G) are also shown.

At the time of laying (day 3), in 2012 we found a negative relationship between number of unpigmented feathers and mesophilic bacterial load on eggshells (Beta(SE) = -0.167(0.089), F = 3.73, df = 1,50, P = 0.068; Fig 2A). However, in 2013 unpigmented feathers did not affect mesophilic bacterial load (F = 0.70, df = 1,60, P = 0.407). In this year, mesophilic bacterial load increased as the season progressed (Beta(SE) = 0.025(0.009), F = 7.48, df = 1,60, P = 0.008; Fig 2B). No other variables affected mesophilic bacterial load at the time of laying in 2012 (F < 1.91, df = 1,49, P > 0.173) and 2013 (F < 0.69, df = 1,60, P > 0.407).

At the end of incubation (day 12), the reduced model showed that eggshells of experimental nests with pigmented feathers treatment harboured lower mesophilic bacterial load (Table 3; Beta(SE) = -0.182 (0.088), t = -2.07, P = 0.042; Fig 2C). However, experimental feather manipulation in 2012, and manipulation of aromatic plant material in 2012 and 2013, did not significantly affect mesophilic bacterial load on the eggshell at the end of incubation (see S2 Appendix).

**Table 3:** Results from GLM explaining mesophilic bacterial density on incubated spotless starling eggshells at the end of incubation (day 12).

	Beta (SE)	df	F	P
2012				
**log pigmented feathers (2**^**nd**^**)**	**-0.151 (0.070)**	**1,50**	**4.74**	**0.034**
**log unpigmented feathers (2**^**nd**^**)**	**0.196 (0.069)**	**1,50**	**8.07**	**0.006**
2013				
**date of first sampling (1 = 1 April)**	**0.015 (0.008)**	**1,57**	**3.59**	**0.063**
**log unpigmented feathers (1**^**st**^**)**	**0.150 (0.072)**	**1,57**	**4.41**	**0.040**
**log pigmented feathers (2**^**nd**^**)**	**0.153 (0.066)**	**1,57**	**5.40**	**0.023**
log unpigmented feathers (2^nd^)	-0.127 (0.070)	1,57	3.26	0.076
Feather treatment		2,57	2.38	0.101

Nest lining materials (pigmented and unpigmented feathers) before incubation started (1^st^) and at the end of incubation (2^nd^) were included as continuous independent factors. Experimental treatments of aromatic plants (with or without) and of feathers (pigmented, unpigmented and without feathers) were included as fixed factors. In 2013, we used a third experimental treatment that consisted on eggshell contamination at the time of egg laying. Interactions between treatments did not reach statistical significance (2012: P = 0.23; 2013: P > 0.15) and are not shown. We only show final models with retained factors with p-values < 0.1. However, associated statistical significance of different factors did not change in full models (see S2 Appendix). Significant associations are in bold.

Feather nest material did also affect mesophilic bacterial load at the end of incubation in both years. In 2012, mesophilic bacterial load was positively related to number of unpigmented feathers at the end of incubation (Table 3; Fig 2D) and negatively related to number of pigmented feathers at the end of incubation (Table 3; Fig 2E). In 2013, mesophilic bacterial load was higher in nests with more unpigmented feathers at time of laying (Fig 2F), and tended to be negatively and positively related to number of unpigmented and pigmented feathers at day 12, respectively (Table 3; Fig 2G). Finally, in 2013 coating eggshells with a solution of bacteria on egg contents did not affect mesophilic bacterial loads (S2 Appendix).

**Fig 3:**
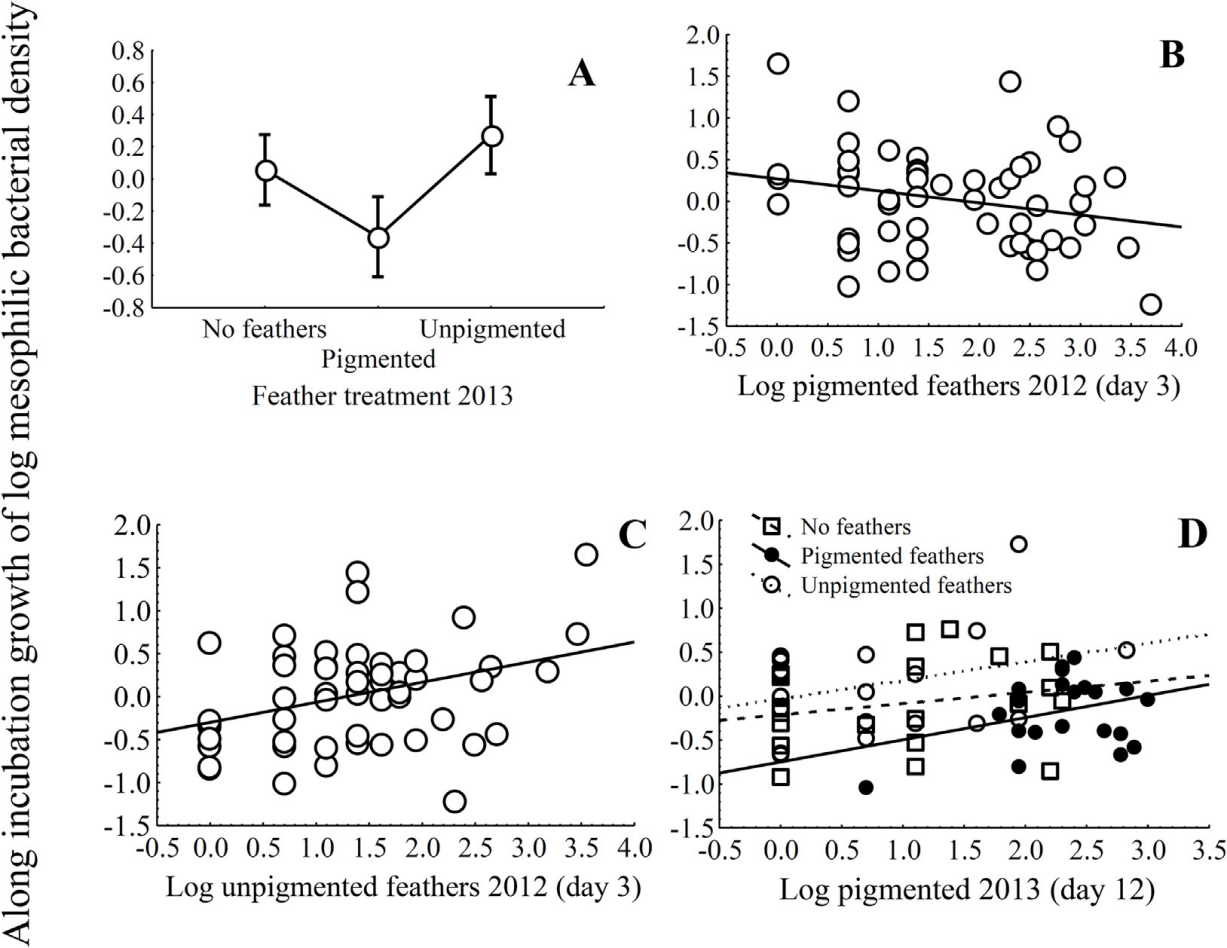
Results of mesophilic bacterial load along incubation (variation between day 3 and day 12). Average values of eggshell mesophilic bacterial growth (± 95% CI) on starling eggshells in relation to feathers’ experimental treatments (unpigmented, pigmented or without feathers) in 2013 (A, D). The associations of eggshell mesophilic bacterial growth with number of pigmented (B) and unpigmented (C) feathers in starling nests in 2012 are also shown. Figure only shows the most relevant associations detected.

When we explored the variation in eggshell bacterial loads along incubation (variation between day 3 and day 12) we found that in 2012, the performed experiments with nest lining materials (aromatic plants or feathers) did not affect changes in mesophilic bacterial loads of eggshells along the incubation period (S3 Appendix). In 2013, experimental manipulation of nest lining feathers, but not that of aromatic plants, did explain changes in mesophilic bacterial loads along incubation period (Table 4): only experimental nests with pigmented feathers, but not nests without feathers or those with unpigmented feathers, did reduce eggshell bacterial loads from laying to the end of incubation (Fig 3A).

**Table 4:** Results from GLM explaining changes in mesophilic bacterial density on spotless starling eggshells along the incubation period (changes between day 3 and day 12).

	Beta (SE)	df	F	P
2012				
**log pigmented feathers (1**^**st**^**)**	**-0.202 (0.077)**	**1,50**	**6.85**	**0.012**
**log unpigmented feathers (1**^**st**^**)**	**0.305 (0.085)**	**1,50**	**11.17**	**0.002**
2013				
**log pigmented feathers (2**^**nd**^**)**	**0.216 (0.082)**	**1,60**	**6.91**	**0.011**
**Feather treatment**		**2,60**	**5.36**	**0.007**

Nest lining material (pigmented and unpigmented feathers) before incubation started (1^st^) and few days before hatching (2^nd^) were included as continuous independent covariates. Experimental modification of green plants (i.e. with or without aromatic plants) and of feathers (i.e. pigmented, unpigmented or without feathers treatment) were included as factors with fixed effects. In 2013, we used a third experimental treatment that consisted on eggshell contamination at the time of egg laying. We only show final models with retained factors with p-values < 0.1. However, associated statistical significances of different factors did not change in full models (see S3 Appendix). Significant associations are in bold.

Variation in eggshell bacterial loads along incubation in 2012 were however explained by nest materials at day 3 (i.e., negatively related with number of pigmented feathers (Fig 3B), and positively related with number of unpigmented feathers (Fig 3C)) (Table 4). In 2013, the final model did retain the number of pigmented feathers at the time of second sampling, which was positively related with change in mesophilic bacterial load (Fig 3D).

#### Nest material and experimental treatments effects on bacteria in specific media (*Enterobacteriaceae*, *Enterococcus and Staphylococcus*)

At time of laying (day 3), prevalence of bacteria in specific media was relatively low, and did not differ between years for *Enterobacteriaceae* (2012 = 9.43%, N = 53; 2013: 7.81%, N = 64, Fisher-exact test; P = 0.75) or *Enterococcus* (2012 = 18.87%, N = 53; 2013: 10.94%, N = 64, Fisher-exact test: P = 0.29). However, prevalence of *Staphylococcus* was higher in 2012 (15.09%, N = 53) than in 2013 (0%, N = 64) (Fisher-exact test: P = 0.0013). Because of the low prevalence of *Staphylococcus* in 2013, we did not explore its association with considered factors.

**Fig 4:**
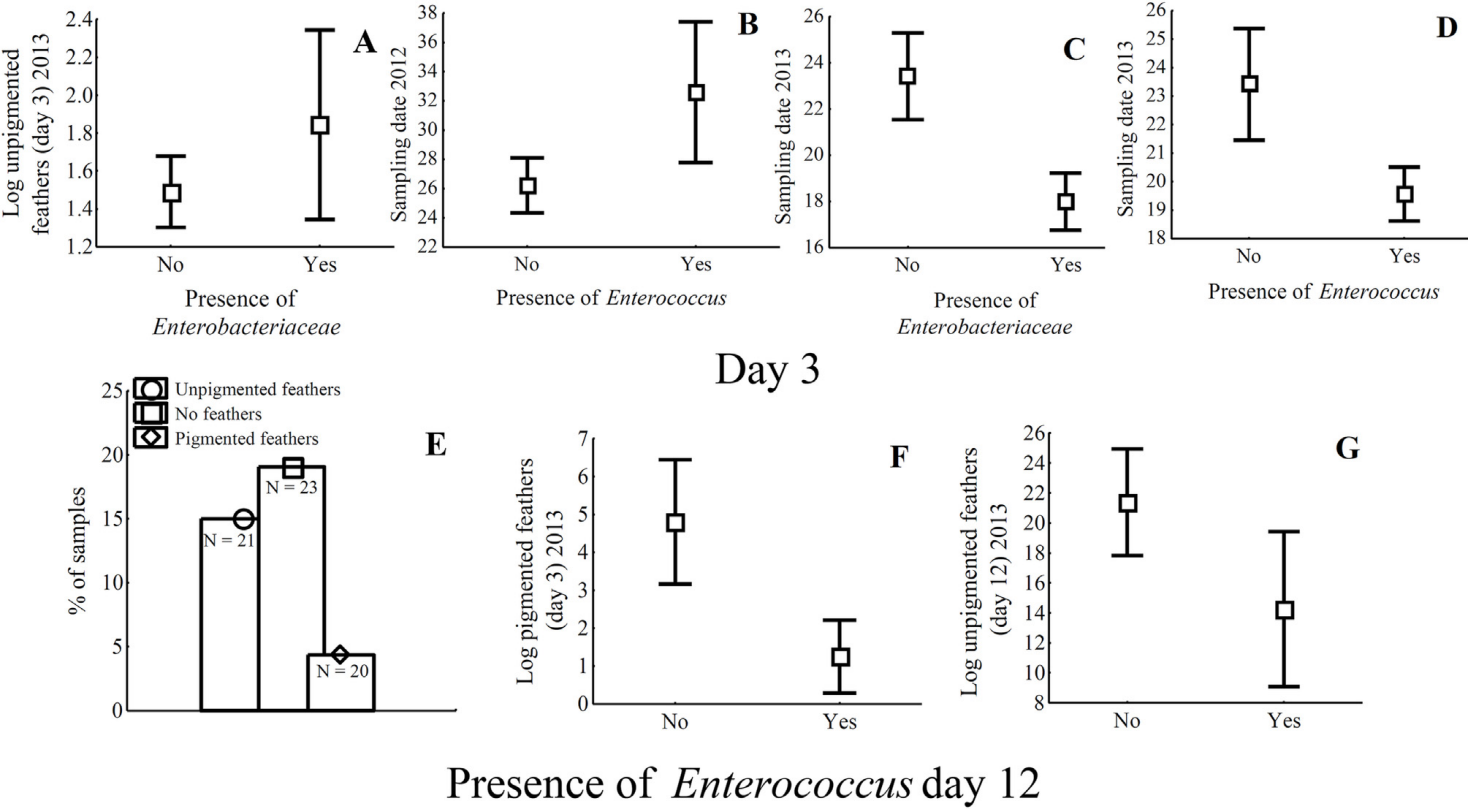
Nest material and experimental treatments effects on bacteria in specific media. Average number (± 95% CI) of unpigmented feathers at day 3 in 2013 in relation to prevalence of *Enterobacteriaceae* (A), and effects of sampling date in relation to prevalence of *Enterococcus* (in 2012, B), *Enterobacteriaceae* (in 2013, C), and *Enterococcus* (in 2013, D). Prevalence of *Enterococcus* at day 12 in relation to feather treatment (in 2013, E), number of pigmented feathers at day 3 (in 2013, F) and number of unpigmented feathers at day 12 (in 2013, G) is also shown. Figures show the most relevant associations detected.

When we explored the effect of nest material at time of laying on bacteria prevalence, we found that number of unpigmented feathers was positively related with *Enterobacteriaceae* presence in 2013 (χ^2^ = 7.58, df = 1, P = 0.006; Fig 4A) and tended to be positively related with *Enterococcus* presence in 2013 (χ^2^ = 2.97, df = 1, P = 0.085).

Nests with higher number of pigmented feathers tended to have lower prevalence of *Enterobacteriaceae* in 2012 (χ^2^ = 3.67, df = 1, P = 0.055), but it did not affect other kind of bacteria in 2012 or 2013 (χ^2^ < 1.61, df = 1, P > 0.205).

As the season progressed, prevalence of *Enterococcus* in 2012 increased (χ^2^ = 6.37, df = 1, P = 0.012; Fig 4B). However, prevalence of *Enterobacteriaceae* and *Enterococcus* in 2013 were lower in late laying nests (*Enterobacteriaceae*: χ^2^ = 18.43, df = 1, P < 0.0001; Fig 4C; *Enterococcus*: χ^2^ = 5.13, df = 1, P = 0.023; Fig 4D). Sampling date was not significantly related to prevalence of *Enterobacteriaceae* or *Staphylococcus* in 2012 (χ^2^ < 2.30, df = 1, P > 0.129).

At the end of incubation (day 12), prevalence of bacteria in specific media were very low and no year differences were found for *Enterobacteriaceae* (2012: 1.83%, N = 53; 2013: 3.13%, N = 64), *Enterococcus* (2012: 3.77%, N = 53; 2013: 2.50%, N = 64; P = 0.101) and *Staphylococcus* (2012: 0%, N = 53; 2013: 1.56%, N = 64) (Fisher-exact tests: P > 0.99). Because of the very low prevalence of specific bacteria groups in samples from incubated eggs in 2012 and *Enterobacteriaceae* and *Staphylococcus* in 2013, we did not explore its association with considered factors or variation along incubation (variation between day 3 and 12).

At the end of incubation, the experimental manipulation of feather nest material had an effect on *Enterococcus* prevalence in 2013. The reduced model showed that eggshells of experimental nests with pigmented feathers treatment had lower *Enterococcus* prevalence than those in nests without feathers or with unpigmented feathers (χ^2^ = 7.25, df = 2, P = 0.027; Fig 4E). However, experimental manipulation of aromatic plant material did not significantly affect *Enterococcus* prevalence in 2013 (complete model: χ^2^ = 0.41, df = 1, P = 0.52).

Nest feather material did also affect *Enterococcus* bacteria in 2013. The reduced model showed that nests with higher number of pigmented feathers at time of laying (χ^2^ = 80.4, df = 1, P = 0.005; Fig 4F) and with higher number of unpigmented feathers at the end of incubation (χ^2^ = 6.30, df = 1, P = 0.012; Fig 4G) were those with the lowest *Enterococcus* prevalence.

### Eggshell bacterial loads in artificial nests with no incubation activity

**Fig 5:**
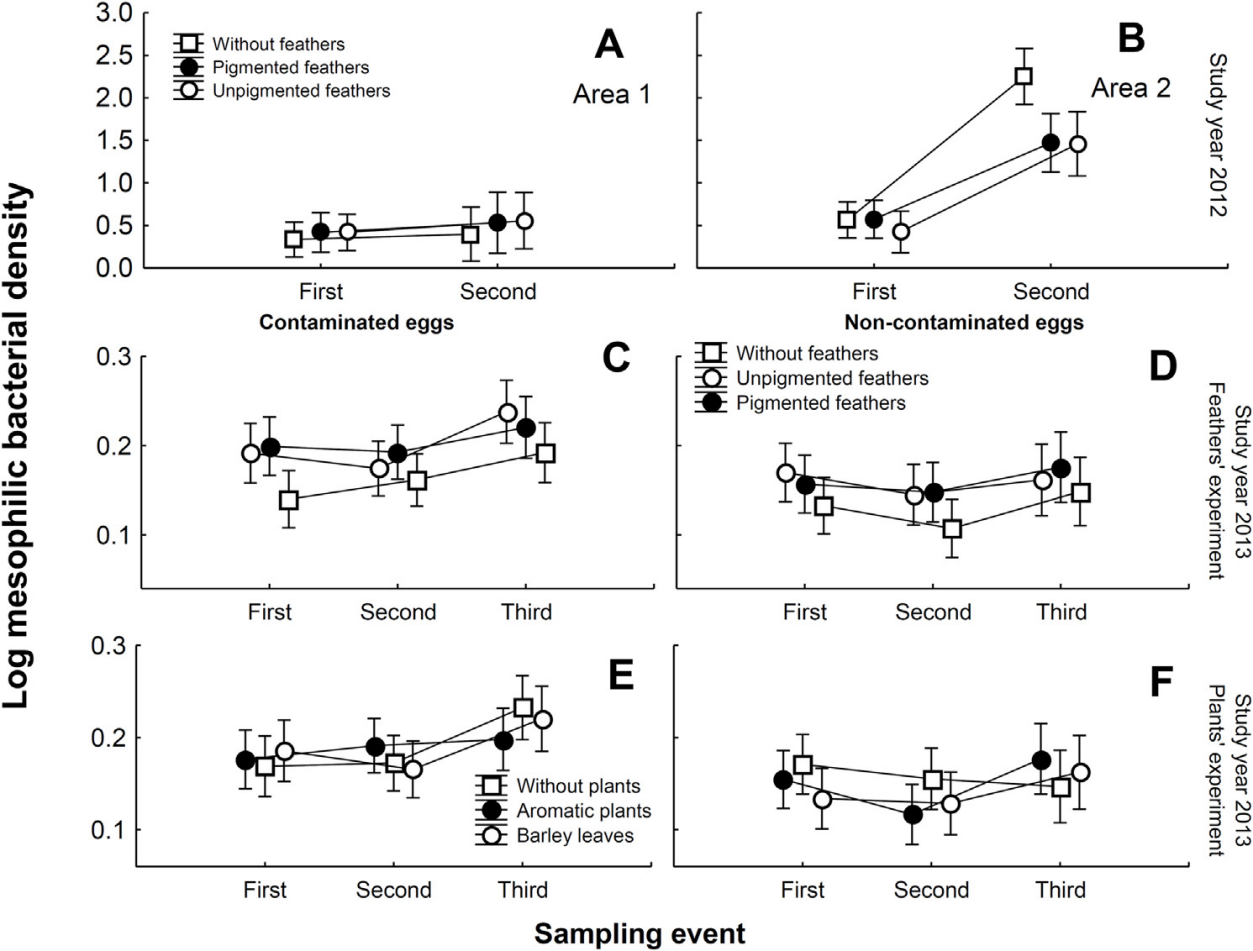
Results of mesophilic bacterial density of quail eggs. Eggshell mesophilic bacterial density (± 95% CI) estimated for experimental quail eggs during the two sampling events in relation to feathers experimental treatment and area for samples collected in 2012 (A and B). The effects of feathers (C and D) and plants (E and F) experimental treatments for contaminated and non-contaminated quail eggs during the three sampling events in 2013 are shown.

In 2012, mesophilic bacterial loads on quail eggshells increased from first to second sampling, mainly for the study area number 2 (Table 5). The effects of experimental feathers on density and growth of mesophilic bacterial loads were apparent for samples from area number 2, but not for those from area number 1 (see interactions between study area and feather treatment, and between sampling events, study area and feather treatment in Table 5). Eggs in nest boxes with pigmented or unpigmented feathers treatments harbored lower density of bacteria than eggs in nests without feathers (Fig 5). Post-hoc analyses did not reveal differences in the effects of pigmented and non-pigmented feathers on eggshell bacterial loads (Fisher LSD; area number 1: P = 0.942, area number 2: P = 0.635) or bacterial growth (area number 1: P = 0.926, area number 2: P = 0.616).

**Table 5:** Results from Repeated Measures ANOVAs explaining mesophilic bacterial loads on quail eggshells.

	F	df	p
Study year: 2012			
Between effects			
**Area (1)**	**53.94**	**1, 67**	**< 0.0001**
Feathers' treatment (3)	1.37	2, 67	0.26
**(1) x (3)**	**4.07**	**2, 67**	**0.021**
Within effects (Repeat measures–sampling)			
**(Sampling)**	**78.56**	**1, 67**	**< 0.0001**
**Sampling x (1)**	**55.59**	**1, 67**	**< 0.0001**
Sampling x (3)	2.42	2, 67	0.097
**Sampling x (1) x (3)**	**3.33**	**2, 67**	**0.042**
Study year: 2013			
Between effects			
**Area (1)**	**97.37**	**1, 54**	**< 0.0001**
Aromatic plants' treatments (2)	0.27	2, 54	0.764
**Feathers' treatment (3)**	**6.00**	**2, 54**	**0.004**
First within effects (Repeat measures—Contamination)			
**(Contamination)**	**22.56**	**1, 54**	**< 0.0001**
Contamination x (1)	0.50	1, 54	0.483
Contamination x (2)	0.27	2, 54	0.764
Contamination x (3)	0.11	2, 54	0.900
Second within effects (Repeat measures–Sampling)			
**(Sampling)**	**6.42**	**2, 108**	**0.002**
Sampling x (1)	1.83	2, 108	0.165
Sampling x (2)	0.15	4, 108	0.961
Sampling x (3)	0.23	4, 108	0.920
First x Second within effects (Repeat measures)			
(Sampling x Contamination)	1.88	2, 108	0.157
Sampling x Contamination x (1)	0.24	2, 108	0.786
**Sampling x Contamination x (2)**	**3.20**	**4, 108**	**0.016**
Sampling x Contamination x (3)	0.96	4, 108	0.432

Quail eggs in experimental nest-boxes without incubation activity were subjected to two different treatments (plants (aromatic plants, non-aromatic plants or no plants) and feathers (pigmented, unpigmented or no feathers) as nest lining materials) in a full factorial design. The experiments were performed in two different areas and two different years. Samples were collected 5, 9 and 17 (only in 2013) days after the onset of the experiment. Thus, the models included study area and experimental treatments as between factors and sampling events and its interaction with study area and experimental treatments as within factors. In 2013, we included an additional within nest experimental treatment consisting on contaminating eggshells in the nests and, thus, contamination and the interaction with sampling event were included as additional within nest effects (repeated measures). We show final models that only include between-factors that alone or in interaction with other variables did result associated with eggshell bacterial loads, at least partially (P < 0.1). Statistically significant factors did not differ from those shown in the full models. Significant associations are in bold.

Experiments with green plants did not affect eggshell bacterial loads or growth during the study period in any of the study areas (S4 Appendix). Finally, we did not find evidence of the interaction between green plants’ and feathers’ experiments determining eggshell bacterial loads (S4 Appendix).

In 2013, we also found significant statistical differences between study areas and among experimental treatments (Table 5). However, the detected effects in this year were contrary to those detected in 2012. Bacterial density of quail eggs was higher in nests with feathers than in nests without feathers (Fig 5). The effect of feather experiment in this case did not depend on the study area (S4 Appendix). Moreover, we found the expected effects of experimental contamination with pathogenic bacteria, which was independent of experimental treatment and study area (Table 5). We also detected an increase in eggshell bacterial loads from first to third samples (Table 5). The interaction between sampling time and contamination experiment only differed for nests under different green plants treatments (Table 5). Eggshell bacterial loads of nests without plants increased from first to third sampling date in experimentally contaminated eggs but decreased in non-contaminated eggs (Fig 5).

## Discussion

Our experimental modification of nest material in starling nests and in artificial nest boxes did affect nest bacterial environments as estimated as eggshell bacterial loads. The expected associations between eggshell bacterial loads and nest materials were most obvious for nest lining feathers’ than for green plants’ experiments. Although some of our bacterial quantifications do not distinguish between potentially pathogenic and non-pathogenic bacteria (i.e., mesophilic bacteria), we also detected evidence of expected associations for *Enterobacteriaceae* and *Enterococcus*, two groups of bacteria that include embryo pathogens. Below we discuss these results that varied depending on the study year and location under the hypothesis that nest lining feathers and green plants have antimicrobial functions.

Considering that transporting feathers and green plants to the nest are costly activities in terms of time and energy [54], birds should adjust these efforts to the expected beneficial consequences. The expected beneficial effects of these nest materials on eggshell bacterial loads were only detected in particular study years and locations, especially those for which high bacterial densities were detected (2013 in starling nests and in artificial nest boxes sampled in the area 2 in 2012). Bacterial loads in starling nests were higher in 2013 than in 2012 and, in accordance with a possible nest building effort adjustment to bacterial environment, starlings carried to the nest more pigmented feathers in 2013 that in 2012 (see Results). Several clues may be used by adults to infer future risk of bacterial proliferation in their nests and accordingly adjust the effort dedicated to collect and transport nest materials with antimicrobial activity. We know for instance that humidity [46,55], temperature [56,57], and characteristics related to laying date [55,58] are good predictors of bacterial growth. Thus, birds may adjust amount and composition of nest materials to environmental conditions, which we found to differ between study years in terms of temperature and humidity. Our results fit at least partially this possibility since amount of antimicrobial nest material detected in starling nests before incubation started predicted the risk of infection during the incubation period, as well as eggshell bacterial loads soon before egg hatching, independently of experimental treatment.

Nest bacterial environments and thus risk of embryo infection also depend on other factors that may directly or indirectly be related to nest building material. Nest building activity has a sexually selected component in birds [59,60]. Particularly for spotless starlings, there is experimental evidence that the use of feathers and green plants as nest material is sexually selected [37,45,61,62]. Thus, it is possible that the detected associations between nest materials and eggshell bacterial loads were a by-product of adult phenotypic characteristics implicated in sexual selection [63].

Incubation activity has also been suggested to have an important antimicrobial function [18,46,55], which may be positively related to the expression of sexually selected characters [64,65]. The bacterial clearance effect of incubation was clearly pointed out with the contamination experiment performed in artificial and natural nests. The detected experimental effect of eggshell contamination on bacterial density in non-incubated artificial nests was counteracted in incubated starling eggs (Table 5). A very similar experiment (eggshell contamination) was recently performed in natural and artificial nests of black billed magpies (*Pica pica*) with exactly the same results [55], therefore, confirming the antimicrobial effects of avian incubation. Thus, the possible interaction between amount of nest lining materials and incubation efficiency of adults reducing eggshell bacterial colonization and growth may explain our findings of bacterial environment modification. We take advantage of experimental results including nest boxes with and without incubation activity to conclude that even assuming a sexually selected component of the studied nest lining materials, these materials have independent effects on bacterial colonization and/or proliferation on starling eggshells.

The antimicrobial effects of particular aromatic plants have been experimentally demonstrated in nests of European starlings (*Sturnus vulgaris*) [30] and on skin of nestling blue tits (*Cyanistes caeruleus*) [32] but never in egg microbiota [66]. We here did not detect such expected effects in nests of spotless starlings. Thus, we cannot discard that the relatively soft manipulations performed made difficult, or was not adequate, to detect antimicrobial effects of aromatic plants in nests of spotless starlings. Another possible explanation is related to the large number of antimicrobial defense lines of natural avian nests against bacterial proliferation on the eggshells and trans-shell infection of embryos [66]. Absence of one of these lines of defense (i.e., green plants) will provoke slight negative effects, possibly requiring large sample sizes to be detected. In accordance with this possibility, we detected the expected effects of experimental green plants in artificial nests with no incubation activity, bacterial growth of previously contaminated experimental eggs that were in contact with aromatic plants was lower than that of eggs in nest boxes without aromatic plants (Table 5) (see also [34]). Thus, we found experimental support for the antimicrobial effects of aromatic plants that would be more difficult to detect in natural nests.

Antimicrobial function of nest lining feathers has recently been suggested, but support for the hypothesis has only been detected in barn swallow (*Hirundo rustica*) nests [41,42,44]. Here, we found experimental and correlative support to the hypothesis in nests of spotless starlings. Previous studies dealing with lining feathers of swallow nests, as well as theoretical work [14], suggested a relatively larger antimicrobial beneficial effect for unpigmented feathers. We here find out experimental support in natural nests for pigmented feathers, but not for unpigmented feathers. Moreover, the amount of pigmented feathers in spotless starling nests at different stages did result negatively related with eggshell bacterial loads and growth more frequently than that of unpigmented feathers, which also suggests larger effects for pigmented feathers. As we discussed for green plants, the detected associations may be a by-product of antimicrobial capability of birds and sexually selected traits (see above). However, this is also unlikely for feathers because the expected antimicrobial effect of nest lining feathers was more clearly detected in nest boxes with no incubation activity. In this case pigmented and unpigmented feathers produced similar effects.

The strength of the experimental effects and even the sign of the detected associations between nest lining materials and eggshell bacterial loads did greatly varied for different statistical models tested. It may simply be the consequence of partial effects in statistical models where independent factors share covariance with the dependent factor (eggshell bacterial loads). Another possibility is that detectable effect of particular nest material (i.e., unpigmented feathers) depends on whether or not other materials (pigmented feathers, or green plants) were present in the nest. We know for instance that bacteria isolated from unpigmented nest lining feathers have higher antimicrobial capabilities if collected from nests that at the beginning of incubation did only contain unpigmented feathers [44]. Thus, it is possible that particular compositions of nest lining feathers select for beneficial bacteria with different antimicrobial capacities. Even more, some of our bacterial quantifications do not distinguish between potentially pathogenic and non-pathogenic bacteria, and some of the detected bacteria on the eggshells may be from nest lining material with the highest bacterial growth (i.e. unpigmented feathers, see Introduction). We predicted a possible effect of green plants on the antimicrobial properties of nest lining feathers and found no support, even in artificial nests without incubation. Thus, although more research is necessary before reaching firm conclusions, we concluded that this interaction is unlikely.

Summarizing, all these results considered together confirm an association between nest materials and bacterial environments of nests that depended on environmental conditions of different study years and localities. Particularly interesting is the association between variations in bacterial environments and in expected effects of nest lining materials, which suggests that birds should adjust nest building effort to bacterial environments. Finally, we hope that the detected experimental effects of feathers as antimicrobial material in avian nests encourage further research looking for mechanisms mediating such effects, including selection of bacterial strains with particular antimicrobial properties depending on nest lining material composition.

## Supporting Information

S1 Appendix Samples sizes for artificial and natural nests in 2012 and 2013. Sample sizes for artificial non-incubated nests with quail eggs and for experimental starling nests under different experimental treatments during 2012 and 2013. Non-incubated nests were subjected to three different experiments: (1) eggshell contamination (contaminated (C) or not (NC) with egg contents with bacteria), (ii) feather experiment (with pigmented, unpigmented, or without (control) feathers) and (iii) plant experiment (with aromatic and non-aromatic plant and with no plants). Starling nests were subjected to the same feather’s experimental treatments, but the plants treatment included only two groups (with and without aromatic plants).
(PDF)

S2 Appendix Results from GLM explaining mesophilic bacterial density on incubated eggshells of spotless starlings few days before hatching (day 12). Nest lining material (pigmented and unpigmented feathers) before incubation started (1^st^) and few days before hatching (2^nd^) were included as continuous independent factors. Experimental modification of green plants (i.e. with or without aromatic plants) and of feathers (i.e. pigmented, unpigmented and without feathers) were included as fixed effects. In 2013, we used a third experimental treatment that consisted on eggshell contamination at the time of egg laying. The interactions between experiments were far from statistical significance (2012: P = 0.23; 2013: P > 0.15) and are not shown. Reduced models show retained factors in the models with p-values < 0.1. Significant relationships are in bold.
(PDF)

S3 Appendix Results from GLM explaining changes in mesophilic bacterial density on eggshells of spotless starlings along the incubation period (variation between day 3 and day 12). Nest lining material (pigmented and unpigmented feathers) before incubation started (1^st^) and few days before hatching (2^nd^) were included as continuous independent factors. Experimental modification of green plants (i.e. with or without aromatic plants) and of feathers (i.e. pigmented, unpigmented and without feathers treatment) were included as fixed effects. In 2013, we used a third experimental treatment that consisted on eggshell contamination at the time of egg laying. The interactions between experiments were far from statistical significance (2012: P > 0.88; 2013: P > 0.10) and are not shown. Significant relationships are in bold.
(PDF)

S4 Appendix Results from Repeated Measures ANOVAs explaining mesophilic bacterial loads of quail eggs in experimental nest-boxes without incubation activity. The artificial nests were subjected to two different experiments: plants (aromatic plants, non-aromatics plants or no plants) and feathers (pigmented, unpigmented or no feathers) as nest lining materials in a full factorial design. The experiments were performed in two different areas and two different years. Samples were collected 5, 9 and 17 (only in 2013) days after the onset of the experiment. Thus, the models included study area and experimental treatments as between factors and sampling events and its interaction with study area and experimental treatments as within factors. In 2013, we included an additional within nest experimental treatment consisting on contaminating some eggshells in the nests and, thus, contamination and the interaction with sampling event were included as additional within nest effects (repeated measures). We show full models. Significant relationships are in bold.
(PDF)
