# Supplementary material for: Nest Material Shapes Eggs Bacterial Environment
Source: PLoS One. 2016 Feb 12;11(2):e0148894. doi: 10.1371/journal.pone.0148894 (PMC4752222; doi:10.1371/journal.pone.0148894)
Supplement: S1 Appendix — Sample sizes for artificial non-incubated nests with quail eggs and for experimental starling nests under different experimental treatments during 2012 and 2013. Non-incubated nests were subjected to three different experiments: (1) eggshell contamination (contaminated (C) or not (NC) with egg contents with bacteria), (ii) feather experiment (with pigmented, unpigmented, or without (control) feathers) and (iii) plant experiment (with aromatic and non-aromatic plant and with no plants). Starling nests were subjected to the same feather’s experimental treatments, but the plants treatment included only two groups (with and without aromatic plants). (PDF) [file pone.0148894.s001.pdf]

S1 Appendix. Samples sizes of artificial and natural nest in 2012 and 2013.

		2012			2013							Total
Non-incubated nest-boxes												
Study area		Pin	Poc	Tot	Pin		CH				Tot	
Contamination treatments		NC	NC		C	NC	C	NC	Tot-C	Tot-NC		
Feather treatments	Plant treatments											
Control	Control	5	4	9	4	8	3	6	7	14	21	30
Control	Non-aromatic	5	4	9	3	5	3	5	6	10	16	25
Control	Aromatic	4	5	9	4	6	4	6	8	12	20	29
Pigmented	Control	2	3	5	4	6	3	5	7	11	18	23
Pigmented	Non-aromatic	4	5	9	5	5	2	4	7	9	16	25
Pigmented	Aromatic	5	4	9	4	6	3	5	7	11	18	27
Unpigmented	Control	3	2	5	4	6	2	3	6	9	15	20
Unpigmented	Non-aromatic	5	5	10	3	4	4	6	7	10	17	27
Unpigmented	Aromatic	5	3	8	3	4	3	5	6	9	15	23
Total	Total	38	35	73	34	50	27	45	61	95	156	229
Starling nests												
Control	Plants			9							11	20
Control	Control			8							10	18
Pigmented	Plants			9							12	21
Pigmented	Control			6							11	17
Unpigmented	Plants			11							11	22
Unpigmented	Control			10							9	19
Total	Total			53							64	117

Sample sizes for artificial non-incubated nests with quail eggs and for experimental starling nests under different experimental treatments during 2012 and 2013. Non-incubated nests were subjected to three different experiments: (1) eggshell contamination (contaminated (C) or not (NC) with egg contents with bacteria), (ii) feather experiment (with pigmented, unpigmented, or without (control) feathers) and (iii) plant experiment (with aromatic and non-aromatic plant and with no plants). Starling nests were subjected to the same feather's experimental treatments, but the plants treatment included only two groups (with and without aromatic plants).
