# Supplementary material for: Nest Material Shapes Eggs Bacterial Environment
Source: PLoS One. 2016 Feb 12;11(2):e0148894. doi: 10.1371/journal.pone.0148894 (PMC4752222; doi:10.1371/journal.pone.0148894)
Supplement: S2 Appendix — Nest lining material (pigmented and unpigmented feathers) before incubation started (1st) and few days before hatching (2nd) were included as continuous independent factors. Experimental modification of green plants (i.e. with or without aromatic plants) and of feathers (i.e. pigmented, unpigmented and without feathers) were included as fixed effects. In 2013, we used a third experimental treatment that consisted on eggshell contamination at the time of egg laying. The interactions between experiments were far from statistical significance (2012: P = 0.23; 2013: P > 0.15) and are not shown. Reduced models show retained factors in the models with p-values < 0.1. Significant relationships are in bold. (PDF) [file pone.0148894.s002.pdf]

S2 Appendix. Results from GLM explaining mesophilic bacterial density on incubated eggshells of spotless starlings few days before hatching (day 12).

2012	Beta (SE)	df	F	P
Date of first sampling (1=1 April)	-0.008 (0.008)	1,44	0.86	0.358
log number of pigmented feathers (1st)	-0.051 (0.056)	1,44	0.83	0.366
log number of unpigmented feathers (1st)	0.083 (0.061)	1,44	1.87	0.179
**log number of pigmented feathers (2nd)**	**-0.172 (0.080)**	**1,44**	**4.64**	**0.037**
**log number of unpigmented feathers (2nd)**	**0.189 (0.080)**	**1,44**	**5.61**	**0.022**
Feather treatment		2,44	0.70	0.501
Aromatic plant treatment		1,44	0.060	0.808
2013				
Date of first sampling (1=1 April)	0.014 (0.009)	1,54	2.56	0.115
log number of pigmented feathers (1st)	0.047 (0.054)	1,54	0.79	0.379
**log number of unpigmented feathers (1st)**	0.147 (0.077)	1,54	3.62	0.062
**log number of pigmented feathers (2nd)**	**0.160 (0.066)**	**1,54**	**5.91**	**0.018**
log number of unpigmented feathers (2nd)	-0.117 (0.073)	1,54	2.61	0.112
Eggshell contamination treatment		1,54	1.54	0.219
Feather treatment		2,54	2.10	0.133
Aromatic plant treatment		1,54	1.12	0.294

Nest lining material (pigmented and unpigmented feathers) before incubation started (1st) and few days before hatching (2nd) were included as continuous independent factors. Experimental modification of green plants (i.e. with or without aromatic plants) and of feathers (i.e. pigmented, unpigmented and without feathers) were included as fixed effects. In 2013, we used a third experimental treatment that consisted on eggshell contamination at the time of egg laying. The interactions between experiments were far from statistical significance (2012: $P = 0.23$; 2013: $P > 0.15$) and are not shown. Reduced models show retained factors in the models with p-values < 0.1. Significant relationships are in bold.
