# Supplementary material for: Nest Material Shapes Eggs Bacterial Environment
Source: PLoS One. 2016 Feb 12;11(2):e0148894. doi: 10.1371/journal.pone.0148894 (PMC4752222; doi:10.1371/journal.pone.0148894)
Supplement: S3 Appendix — Nest lining material (pigmented and unpigmented feathers) before incubation started (1st) and few days before hatching (2nd) were included as continuous independent factors. Experimental modification of green plants (i.e. with or without aromatic plants) and of feathers (i.e. pigmented, unpigmented and without feathers treatment) were included as fixed effects. In 2013, we used a third experimental treatment that consisted on eggshell contamination at the time of egg laying. The interactions between experiments were far from statistical significance (2012: P > 0.88; 2013: P > 0.10) and are not shown. Significant relationships are in bold. (PDF) [file pone.0148894.s003.pdf]

S3 Appendix. Results from GLM explaining changes in mesophilic bacterial density on eggshells of spotless starlings along the incubation period (variation between day 3 and day 12).

2012	Beta (SE)	df	F	P
Complete model				
Date of first sampling (1=1 April)	-0.006 (0.012)	1,44	0.24	0.626
**log number of pigmented feathers (1st)**	**-0.214 (0.081)**	**1,44**	**6.97**	**0.011**
**log number of unpigmented feathers (1st)**	**0.255 (0.089)**	**1,44**	**8.26**	**0.006**
log number of pigmented feathers (2nd)	-0.130 (0.116)	1,44	1.26	0.267
log number of unpigmented feathers (2nd)	0.062 (0.116)	1,44	0.29	0.594
Feather treatment		2,44	1.09	0.344
Aromatic plant treatment		1,44	1.81	0.186
2013				
Date of first sampling (1=1 April)	-0.014 (0.012)	1,54	1.34	0.252
log number of pigmented feathers (1st)	0.041 (0.071)	1,54	0.34	0.565
log number of unpigmented feathers (1st)	0.111 (0.103)	1,54	1.16	0.285
**log number of pigmented feathers (2nd)**	**0.221 (0.088)**	**1,54**	**6.33**	**0.015**
log number of unpigmented feathers (2nd)	-0.144 (0.097)	1,54	2.21	0.143
Eggshell contamination treatment		1,54	2.68	0.108
**Feather treatment**		**2,54**	**5.64**	**0.006**
Aromatic plant treatment		1,54	1.23	0.273

Nest lining material (pigmented and unpigmented feathers) before incubation started (1st) and few days before hatching (2nd) were included as continuous independent factors. Experimental modification of green plants (i.e. with or without aromatic plants) and of feathers (i.e. pigmented, unpigmented and without feathers treatment) were included as fixed effects. In 2013, we used a third experimental treatment that consisted on eggshell contamination at the time of egg laying. The interactions between experiments were far from statistical significance (2012: P > 0.88; 2013: P > 0.10) and are not shown. Significant relationships are in bold.
