# Supplementary material for: Nest Material Shapes Eggs Bacterial Environment
Source: PLoS One. 2016 Feb 12;11(2):e0148894. doi: 10.1371/journal.pone.0148894 (PMC4752222; doi:10.1371/journal.pone.0148894)
Supplement: S4 Appendix — The artificial nests were subjected to two different experiments: plants (aromatic plants, non-aromatics plants or no plants) and feathers (pigmented, unpigmented or no feathers) as nest lining materials in a full factorial design. The experiments were performed in two different areas and two different years. Samples were collected 5, 9 and 17 (only in 2013) days after the onset of the experiment. Thus, the models included study area and experimental treatments as between factors and sampling events and its interaction with study area and experimental treatments as within factors. In 2013, we included an additional within nest experimental treatment consisting on contaminating some eggshells in the nests and, thus, contamination and the interaction with sampling event were included as additional within nest effects (repeated measures). We show full models. Significant relationships are in bold. (PDF) [file pone.0148894.s004.pdf]

S4 Appendix. Results from Repeated Measures Anovas explaining mesophilic bacterial loads of quail eggs in experimental nest-boxes without incubation activity.

	Whole model			Reduced model		
2012	F	df	p	F	df	p
Between effects						
**Area (1)**	**54.40**	**1, 55**	**< 0.0001**	**53.94**	**1, 67**	**< 0.0001**
Plants' treatment (2)	0.47	2, 55	0.629			
Feathers' treatment (3)	1.31	2, 55	0.279	1.37	2, 67	0.26
(1) x (2)	0.04	2, 55	0.965			
**(1) x (3)**	**3.86**	**2, 55**	**0.027**	**4.07**	**2, 67**	**0.021**
(2) x (3)	1.95	4, 55	0.115			
(1) x (2) x (3)	1.25	4, 55	0.301			
Within effects (Repeat measures – sampling)						
**(Sampling)**	**71.90**	**1, 55**	**< 0.0001**	**78.56**	**1, 67**	**< 0.0001**
**Sampling x (1)**	**50.71**	**1, 55**	**< 0.0001**	**55.59**	**1, 67**	**< 0.0001**
Sampling x (2)	0.24	2, 55	0.789			
Sampling x (3)	1.81	2, 55	0.174	2.42	2, 67	0.097
Sampling x (1) x (2)	0.12	2, 55	0.890			
**Sampling x (1) x (3)**	**2.73**	**2, 55**	**0.074**	**3.33**	**2, 67**	**0.042**
Sampling x (2) x (3)	1.20	4, 55	0.320			
Sampling x (1) x (2) x (3)	0.82	4, 55	0.521			
2013						
Between effects						
**Area (1)**	**90.87**	**1, 42**	**< 0.0001**	**97.37**	**1, 54**	**< 0.0001**

Aromatic plants' treatment (2)	0.24	2, 42	0.787	0.27	2, 54	0.764
**Feathers' treatment (3)**	**4.69**	**2, 42**	**0.015**	**6.00**	**2, 54**	**0.004**
(1) x (2)	0.24	4, 42	0.914			
(1) x (3)	0.52	2, 42	0.601			
(2) x (3)	2.34	2, 42	0.109			
(1) x (2) x (3)	0.73	4, 42	0.577			
First within effects						
**Repeat measures (Contamination)**	**19.39**	**1, 42**	**< 0.0001**	**22.56**	**1, 54**	**< 0.0001**
Contamination x (1)	0.58	1, 42	0.450	0.50	1, 54	0.483
Contamination x (2)	0.18	2, 42	0.840	0.27	2, 54	0.764
Contamination x (3)	0.07	2, 42	0.934	0.11	2, 54	0.900
Contamination x (1) x (2)	0.66	4, 42	0.624			
Contamination x (1) x (3)	1.30	2, 42	0.284			
Contamination x (2) x (3)	0.32	2, 42	0.726			
Contamination x (1) x (2) x (3)	0.11	4, 42	0.978			
Second within effects						
**Repeat measures (Sampling)**	**5.56**	**2, 84**	**0.005**	**6.42**	**2, 108**	**0.002**
Sampling x (1)	1.44	2, 84	0.243	1.83	2, 108	0.165
Sampling x (2)	0.15	4, 84	0.961	0.15	4, 108	0.961
Sampling x (3)	0.30	4, 84	0.880	0.23	4, 108	0.920
Sampling x (1) x (2)	1.04	8, 84	0.416			
Sampling x (1) x (3)	0.72	4, 84	0.583			
Sampling x (2) x (3)	1.69	4, 84	0.159			
Sampling x (1) x (2) x (3)	0.70	8, 84	0.692			
First x Second within effects						
Repeat measures (Sampling x	1.27	2, 84	0.287	1.88	2, 108	0.157

Contamination)						
Sampling x Contamination x (1)	0.14	2, 84	0.868	0.24	2, 108	0.786
**Sampling x Contamination x (2)**	**2.71**	**4, 84**	**0.036**	**3.20**	**4, 108**	**0.016**
Sampling x Contamination x (3)	0.70	4, 84	0.591	0.96	4, 108	0.432
Sampling x Contamination x (1) x (2)	0.64	8, 84	0.738			
Sampling x Contamination x (1) x (3)	0.80	4, 84	0.531			
Sampling x Contamination x (2) x (3)	0.38	4, 84	0.824			
Sampling x Contamination x (1) x (2) x (3)	0.75	8, 84	0.651			

The artificial nests were subjected to two different experiments: plants (aromatic plants, non-aromatics plants or no plants) and feathers (pigmented, unpigmented or no feathers) as nest lining materials in a full factorial design. The experiments were performed in two different areas and two different years. Samples were collected 5, 9 and 17 (only in 2013) days after the onset of the experiment. Thus, the models included study area and experimental treatments as between factors and sampling events and its interaction with study area and experimental treatments as within factors. In 2013, we included an additional within nest experimental treatment consisting on contaminating some eggshells in the nests and, thus, contamination and the interaction with sampling event were included as additional within nest effects (repeated measures). We show full models. Significant relationships are in bold.
